# Neuropsychiatric Features in Patients With Idiopathic Normal Pressure Hydrocephalus: A Systematic Review and Meta-Analysis

**DOI:** 10.1212/CPJ.0000000000200586

**Published:** 2026-02-05

**Authors:** Clara Belessiotis-Richards, Emma Brady, Esha Abrol, Veronica Chance, Eileen Joyce, Ahmed Toma, Robert Stewart, Gill Livingston

**Affiliations:** 1 Department of Psychological Medicine, Institute of Psychiatry, Psychology & Neuroscience (IoPPN), King's College London, United Kingdom;; 2 Division of Psychiatry, University College London, United Kingdom;; 3 North London NHS Foundation Trust, United Kingdom;; 4 Lived Experience Expert, United Kingdom;; 5 University College London Queen Square Institute of Neurology, United Kingdom; and; 6 National Hospital for Neurology and Neurosurgery, Queen Square, University College London NHS Foundation Trust, United Kingdom.

## Abstract

**Purpose of Review:**

Idiopathic normal pressure hydrocephalus (iNPH) is a treatable condition characterized by impaired gait, cognition, and bladder function. Neuropsychiatric symptoms may be important in iNPH but are poorly understood. We report the first systematic review and meta-analysis estimating the prevalence, severity, and treatment responsiveness of neuropsychiatric symptoms and diagnoses in iNPH. We searched PubMed, CINAHL, Embase, Ovid, MEDLINE, Cochrane, and PsycINFO from inception until October 23, 2024, for peer-reviewed, original studies in adults with definite, probable, or possible iNPH that reported neuropsychiatric features using validated tools or clinician diagnosis (PROSPERO CRD42021287293). Case studies and series, as well as studies where diagnostic criteria for iNPH were unclear, were excluded. Study quality assessment and data extraction were independently performed by 2 authors, according to a proforma developed iteratively. Random-effects meta-analysis was used to pool proportions and standardized mean differences. Meta-regression, subgroup analysis, and sensitivity analysis were performed.

**Recent Findings:**

Twenty-two studies were included, of which 1 was a randomized-controlled trial. For our primary outcome, we found a high prevalence of apathy (69.2%, 95% CI 63.1–74.6, n = 293) and depression (30.1%, 20.1–42.3, n = 7,670) in iNPH. Agitation (22.6%, 11.8–39.1), anxiety (21.9%, 13.2–34.2), disinhibition (21.0%, 11.8–34.7), and psychotic syndromes (8.0%, 3.3–18.3) were relatively less prevalent. Depression scores were higher in patients with iNPH than in controls (Hedge's g 1.31, 0.39–2.23). Treatment of iNPH was associated with a reduction in depression scores (−0.30, −0.62 to 0.01), although the confidence interval contained the null. A total of 12 of 22 studies were rated 'low' quality.

**Summary:**

Apathy and depression are highly prevalent in iNPH, maybe more so than in other neurodegenerative conditions. Little is known about other neuropsychiatric features in iNPH. Treatment of iNPH may reduce neuropsychiatric symptoms, particularly depression. However, our study was limited by heterogeneity in populations and assessment tools and a lack of baseline data on neuropsychiatric symptoms before iNPH diagnosis. These findings highlight the urgent need for further research into neuropsychiatric features in iNPH, their mechanisms, and their potential response to treatment.

## Introduction

Normal pressure hydrocephalus (NPH) was first described in 1965 as a triad of gait disturbance, cognitive impairment, and urinary incontinence in the presence of a communicating hydrocephalus without excessively raised intracranial pressure.^1^ Treatment for NPH includes neurosurgical insertion of a shunt to drain CSF from the ventricles.^2-4^

NPH can occur after triggers such as stroke.^5^ When the NPH syndrome occurs without a clear cause, it is termed “idiopathic” NPH (iNPH).^6,7^ Diagnosis of iNPH is made through a combination of clinical symptoms and characteristic imaging findings according to 2 classification systems: International^8^ and Japanese criteria.^6^ Diagnosis of “probable” iNPH requires evidence of low opening pressure on lumbar puncture, whereas a diagnosis of “possible” iNPH is given if this information is not available. In the Japanese guidelines, “definite” iNPH is diagnosed if there is shunt response.

A multidisciplinary task force reporting on co-occurring conditions found a lack of evidence on the frequency of psychiatric comorbidity in iNPH and highlighted that this might lead to underidentification and undertreatment of psychiatric symptoms.^9^ One clinical cohort estimated that 46% of people treated for iNPH had at least one psychiatric diagnosis.^10^ Patients with iNPH have been found to have frequent depression,^11^ apathy,^12^ psychosis,^13^ and obsessive-compulsive disorder.^14^ In addition, there is evidence of an increased risk of iNPH in schizophrenia.^15^

Psychiatric symptoms including depression are common in other neurodegenerative conditions such as Alzheimer disease (AD)^16^ and Parkinson disease (PD),^17^ which may coexist with iNPH. In other forms of dementia, psychiatric symptoms have been shown to significantly affect quality of life (QOL).^18^ However, the prevalence and severity of psychiatric symptoms in iNPH, and their responsiveness to treatment of iNPH, are not fully understood.

In this first systematic review and meta-analysis, we aim to provide a synthesis of evidence to guide research and clinical practice. Our objectives were to (1) estimate the prevalence of psychiatric features in iNPH, (2) report the severity of psychiatric symptoms in iNPH, and (3) assess the impact of shunting on psychiatric features.

## Methods

This systematic review was prospectively registered (PROSPERO CRD42021287293).^19^ We report our review and abstract according to the Preferred Reporting Items for Systematic Reviews and Meta-Analysis (PRISMA) checklist (eAppendixes 1 and 2).

### Inclusion and Exclusion Criteria

In line with a previous systematic review,^20^ we defined “psychiatric” as features listed under category 06 in the 11th edition of the International Classification of Diseases.^21^ We included peer-reviewed, original studies in adults that reported results on neuropsychiatric features using validated tools, clinician judgment, or medical records.

We only included studies in adults with definite, probable, or possible iNPH, according to either international or Japanese criteria, when this was stated or when sufficient information was offered by authors to determine equivalence. We applied no restrictions on language, setting, or date.

We excluded case studies and series. Studies reporting on patients being assessed for possible iNPH with no breakdown by diagnosis were excluded.

### Search Strategy

We searched PubMed, CINAHL, MEDLINE, APA PsycINFO, Embase, and Cochrane for peer-reviewed publications from inception to November 4, 2021. Searches were rerun on October 23, 2024. The search strategy for iNPH combined the following terms with the Boolean operator OR: “hydrocephalus, normal pressure,” “normal pressure hydrocephalus,” “chronic adult hydrocephalus,” and combinations of “hydrocephalus,” “adult,” “chronic,” “normal,” and “pressure.” For psychiatric symptoms, we combined the following terms with the Boolean operator OR: “psychiatr*,” “mental health,” “neuropsychiatr*,” “depress*,” “anxi*” “apath*,” “mental illness,” “neuro-psychiatric inventory,” “neuropsychiatric inventory,” “NPI,” and “sleep*.” We searched for sleep because disturbance is frequently co-pathologic with neuropsychiatric features, but we only reported symptoms or diagnoses from category 06 of ICD-11. In our final search, we combined iNPH and psychiatric search terms using the Boolean operator AND. All search terms were applied to Medical Subject Headings (if applicable) and to any word in the text (“Text Word”). The full search strategy is included in eAppendix 3. In addition, we manually searched reference lists of included studies for relevant publications.

### Selection Process

Duplicates were determined using automated tools during the search strategy and then through a Python script assessing the first 10 characters of title names.

For the searches performed in 2021, screening of titles and abstracts was conducted by one author (C.B.-R.), with a subset independently screened by a second author (E.A.). Inter-rater reliability was substantial (Cohen kappa = 0.79). For the updated search results, performed in 2024, all titles and abstracts were independently screened by 2 authors (C.B.-R., V.C., E.B.).

All full texts retrieved were reviewed by 2 authors for inclusion (E.A., C.B.-R., E.B.). Disagreements were resolved through discussion with senior authors (G.L., E.J., R.S.). If several studies reported on the same population, we only included the population once.^22,23^ We contacted study authors for clarification in these instances.

### Data Extraction

A data collection proforma was developed (eAppendix 4) and adapted iteratively. Data extraction for full texts was performed by 2 authors independently (E.A., C.B.-R., E.B.). We extracted data on study design, setting, diagnostic criteria used for iNPH, case definition for iNPH (i.e., probable, possible, or definite), participant number, participant characteristics (age, sex), psychiatric assessment tool used, and iNPH severity. Japanese guidelines suggest possible use of a lumbar puncture to estimate response to shunting, known as a “tap test,” as a supportive investigation in assessing probable iNPH. Improvement in symptoms after this has a high positive predictive value (92%)^24^ for shunt responsiveness, though this is rater-dependent.^25^ We extracted data on tap test and shunting, to establish whether neuropsychiatric features varied according to “certainty” of iNPH. We also included data on severity of iNPH using the idiopathic normal-pressure hydrocephalus grading scale (iNPHGS)^26^ and modified Rankin Scale (mRS)^27^ to examine their potential influence on the prevalence and subtype of neuropsychiatric features.

We extracted our outcomes of interest neuropsychiatric feature prevalence and test score results before and after shunting.

### Quality Assessment

Quality and risk-of-bias assessments of full texts were performed by 2 authors independently (C.B.-R., E.A., E.B.) using the Newcastle-Ottawa Scale (NOS).^28^ We then converted overall results to the Agency for Healthcare Research and Quality (AHRQ) standards, which is a standard method for conceptualizing overall quality of a study using NOS.^29^ For randomized-controlled trial data, we used the Risk of Bias-2 tool (RoB 2).^30^ For one Mendelian randomization (MR) study, we used a previously developed tool.^31^

### Analysis

#### Effect Measures

We extracted the proportion with iNPH with each neuropsychiatric symptom or diagnosis before shunting, unless otherwise stated. We extracted effect estimates, primarily odds ratios (ORs), of psychiatric diagnosis compared with people without iNPH. We extracted mean or median scores of symptom severity compared with healthy individuals and after shunting. When insufficient numbers of studies were available for meta-analysis, we reported results narratively.

#### Synthesis Methods

We performed random-effects meta-analysis using R (version 4.5.0) when results were available from at least 2 studies. We used the meta R library for meta-analysis of proportions to estimate the prevalence of neuropsychiatric features. We used the metafor R library for analyses of severity and response to shunting to compute a standardized mean difference (SMD). We converted median and interquartile range to mean and SD using the Luo method.^32^ Heterogeneity was considered important when *I*^2^ was 75% or above.^33^

We explored heterogeneity using meta-regression and subgroup analysis. Sensitivity analysis was performed among studies with higher ‘certainty’ of iNPH (defined as shunted or with a positive tap test result), according to diagnostic criteria as these could be an important source of variation in results and as a combination of certainty and criteria. These were limited to meta-analyses with more than 2 studies.

In our primary analysis, we included the prevalence of any depressive symptoms, including mild. Population screening tools tend to overestimate depressive symptoms relative to clinically relevant depression, so we performed a sensitivity analysis for cutoffs consistent with moderate to severe depression in studies that reported them. We also undertook sensitivity analyses excluding low-quality studies and examining timing of diagnostic criteria.

#### Reporting Bias Assessment

Owing to fewer than 10 studies being available for each outcome of interest, we did not undertake tests of reporting bias.

### Data Availability

All data used in this analysis are published data. Data extracted from included studies, data used for all analyses, and analytic code are all available on request from the lead author C. Belessiotis-Richards.

## Results

### Study Selection

**Figure 1:**
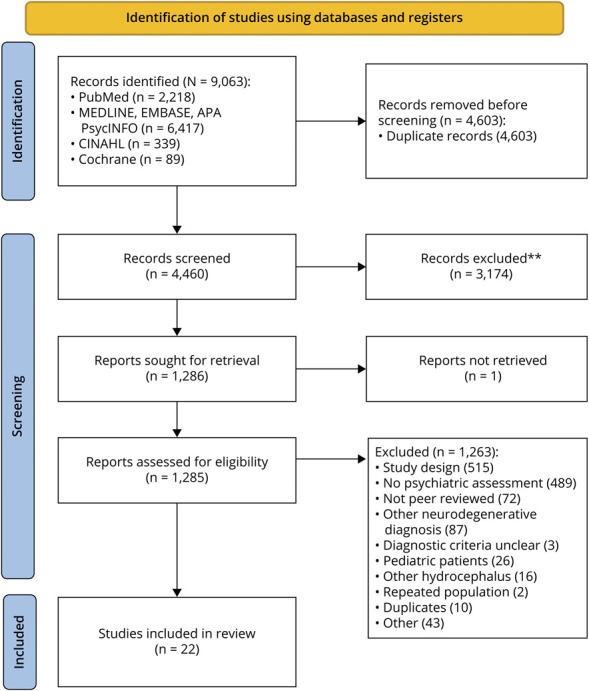
Preferred Reporting Items for Systematic Reviews and Meta-Analysis Diagram

Initial searches in November 2021 found 7,467 articles, with 1,702 additional articles found in October 2024, yielding a total of 9,169 articles (Figure 1). After duplicates, 4,057 unique titles were screened and 1,286 studies were sought for retrieval, of which 1,263 were excluded and one was not available. The most common reason for exclusion was study design. Twelve studies seemed to meet the inclusion criteria but were excluded because of duplication of the population studied,^22,34-36^ a lack of clear diagnostic criteria for iNPH,^37-40^ and a lack of validated psychiatric tools.^41-44^

### Study Characteristics

In total, 22 studies^10-12,23,45-50,e1-e12^ were included in the review (eTable 1). The number of iNPH cases in each study ranged from 5 to 7,199 (in health records). Mean ages of patients with iNPH ranged from 72.2 to 83.0 years, and 15.8% to 59.4% of participants were female. Most studies were cohort or case-control designs, with 1 Mendelian randomization (MR) study^46^ and 1 pilot randomized-controlled trial (RCT).^48^ Studies were predominantly from high-income countries, except 1 study from India. Half of the studies were set in Japan or North America. Date of publication ranged from 2008 to 2024.

Psychiatric symptoms were captured using the Neuropsychiatric Inventory (NPI), Geriatric Depression Scale (GDS and GDS-15), Starkstein Apathy Scale (SAS), Hospital Anxiety and Depression Scale, Beck Depression Inventory (BDI and BDI-II), Cambridge Behavioral Inventory (CBI), Frontal Systems Behavior Rating Scale, Patient Health Questionnaire-9, Yesavage Geriatric Depression Scale, and Mini-International Neuropsychiatric Interview (MINI) (eTable 1). Psychiatric diagnoses were recorded using clinical records of ICD codes and medication use (e.g., antipsychotics for psychotic syndromes). Level of severity was similar across studies reporting this, with the mean or median score ranging from 6 to 6.2 on iNPHGS and from 1.8 to 2.8 on the mRS.

### Risk of Bias

Twelve studies were rated “low” and 8 “good” on the NOS (eAppendix 5). The MR study scored 13/20 on a study-specific assessment scale, and the pilot RCT was rated as having “some concern” of bias.

### Prevalence of Psychiatric Symptoms and Diagnoses in iNPH

Thirteen studies^10-12,23,45,49,e1-e7^ reported on the prevalence of neuropsychiatric features in iNPH, including agitation, anxiety, apathy, depression, disinhibition, and psychotic syndromes. We combined schizophrenia, psychotic syndromes, hallucinations, delusions, and antipsychotic prescription into any psychotic syndrome for meta-analysis. One study^e1^ found overall that 73.4% of patients with iNPH had at least 1 neuropsychiatric symptom on NPI.

#### Apathy

**Figure 2:**
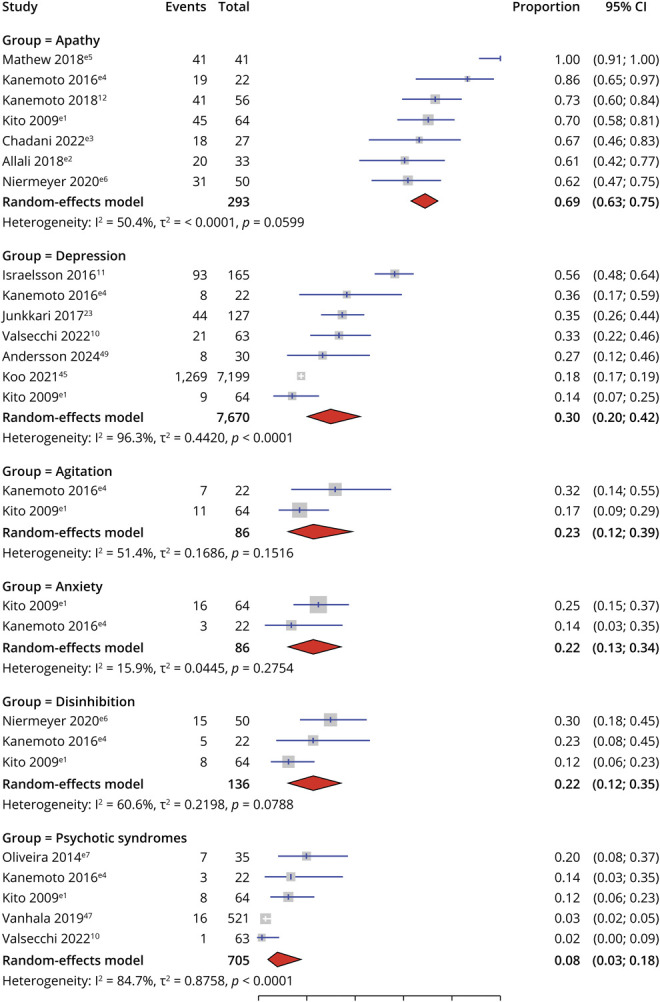
Meta-Analysis of Proportions of Prevalence of Psychiatric Symptoms and Diagnoses in Patients With Idiopathic Normal Pressure Hydrocephalus

Seven case-control or cohort studies^12,^^e1-e6^ reported on the prevalence of apathy (n = 293), with a pooled prevalence of 69.2% (95% CI 63.1–74.6, *I*^2^: 50.4%) (Figure 2). Participant numbers ranged from 22 to 64. One study^e2^ used a tool that solely assessed apathy, the SAS (60.6%). Five studies used the NPI, and 1 used the CBI. All populations were clinical inpatients or outpatients. Three studies were rated as good quality and the rest as low.

#### Depression

Seven case-control or cohort studies^10,11,23,45,49,e1,e4^ reported the prevalence of depression or depressive symptoms (n = 7,670), indicating a pooled prevalence of 30.1% (95% CI 20.1–42.3, *I*^2^: 96.3%) (Figure 2). Participant numbers ranged from 22 to 7,199. Studies reported on clinically diagnosed depression using ICD-9 codes and medical records of antidepressant prescription and on depressive symptoms measured on NPI, GDS-15, and BDI. One study assessed a general population^49^ sample while the rest were clinical participants. Two studies were rated good and the rest low.

A further study^e7^ that was not meta-analyzed in this category reported a prevalence of 48.6% of “depression-anxiety syndrome” in a clinical population (n = 35) in Brazil, using the MINI. This study was rated as low quality.

#### Agitation and Anxiety

Two studies^e1,e4^ reported on the prevalence of agitation (n = 86) and anxiety (n = 86), indicating a pooled prevalence of 22.6% (95% CI 11.8–39.1, *I*^2^: 51.4%) and 21.9% (95% CI 13.2–34.2, *I*^2^: 15.9%) (Figure 2). Both used the NPI, were observational, and included clinical populations in Japan. Quality ratings were low for both.

#### Disinhibition

Three studies^e1,e4,e6^ reported on the prevalence of disinhibition (n = 136), indicating a pooled prevalence of 21.0% (95% CI 11.8–34.7, *I*^2^: 60.6%) (Figure 2). All studies used the NPI and were observational studies involving clinical participants. Two were set in Japan and 1 in the United States. Quality ratings were low for all.

#### Any Psychotic Syndrome

Five studies^10,47,e1,e4,e7^ reported on the prevalence of any psychotic syndromes (n = 705), indicating a pooled prevalence of 8.0% (95% CI 3.3–18.3, *I*^2^: 84.7%) (Figure 2). Two studies^e1,e4^ reported on delusions and hallucinations using the NPI, 1 study^10^ on antipsychotic prescriptions, one study^e7^ on psychotic syndromes using the MINI, and 1 study^47^ on recorded diagnosis of schizophrenia. All studies used clinical populations. Quality ratings were low for all.

### Severity of Psychiatric Symptoms in iNPH

**Figure 3:**
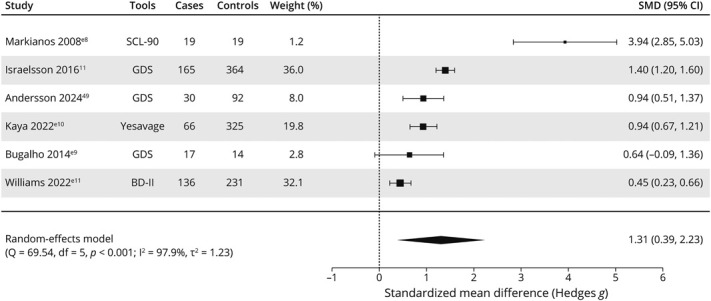
Meta-Analysis of Raw Scores on Depression Scales in Patients With Idiopathic Normal Pressure Hydrocephalus Compared With Controls

Five studies^11,49,e8,e9,e10^ (n = 1,111) reported raw scores on depressive symptom screening tools in patients with iNPH compared with controls, and 1 study^e11^ (n = 367) compared patients with iNPH with people assessed for possible iNPH who were subsequently found not to have iNPH. There were only enough studies for depression meta-analysis. Pooled SMD between people with iNPH and people without iNPH was 1.31 (95% CI: 0.39–2.23, *I*^2^: 97.9%) (Figure 3). Three studies used GDS, and 1 study each used BDI-II, YSGS, and SCL-90. Quality ratings were good for 2 studies and low for 4.

A study by Markianos et al.^e8^ was not included in the meta-analysis but reported significant differences in raw scores compared with controls on phobic anxiety, anxiety, paranoid ideation, and psychoticism domains of the SCL-90.

Results were inconsistent as to whether AD co-pathology influenced results (eTable 2). One study^e12^ found that overall NPI score was higher among AD biomarker–negative than positive iNPH cases, while another^e6^ found higher scores on FrSBE in patients with iNPH with positive AD biomarkers. One study^e3^ found that 11% of patients with iNPH had positive CSF AD biomarkers, regardless of their apathy level, and another^47^ found the same proportion of patients with iNPH with Aβ or hyperphosphorylated tau on cortical biopsy (44–45%), regardless of schizophrenia diagnosis.

### Risk of Psychiatric Diagnoses in iNPH

Two studies^46-47^ reported on the risk of schizophrenia in iNPH. When compared with the general population, 1 study^47^ found increased prevalence of schizophrenia in iNPH, with an absolute risk difference of 2.2% (*p* < 0.001). A MR study^46^ reported on bidirectional risk of iNPH in psychiatric disorders. The authors found that an increased risk of genetic predisposition to schizophrenia was associated with genetic predisposition to iNPH (OR 1.03, 95% CI: 1.01–1.05, *p* = 0.001). These studies were deemed too different to be included in the meta-analysis.

Three studies^10,46,50^ reported on the risk of any psychiatric diagnosis in iNPH. One study^50^found that people with iNPH had 2.48 times higher odds of having a history of recorded psychiatric disorder compared with people without iNPH (95% CI 1.08–5.68, *p* = 0.029). The same study also reported a higher likelihood of a positive depression screen in people with iNPH compared with controls (OR 1.29, 95% CI 0.49–3.38). One study^11^ found an increased adjusted likelihood of mild (OR 8.5, 95% CI 5.5–13.2), moderate (OR 12.3, 95% CI 5.8–26.1), or severe (OR 27.8, 95% CI 6.4–120.2) depression in people with iNPH compared with a general population sample.

The MR study^46^ found no significant association between genetic predisposition to iNPH and genetic predisposition to anxiety, bipolar affective disorder, major depression, or attention-deficit hyperactivity disorder.

### Response to Shunt Insertion Surgery

**Figure 4:**
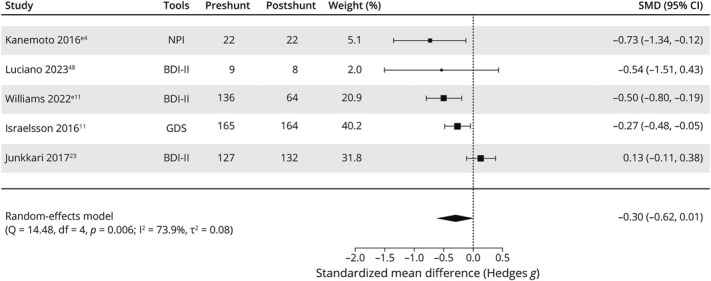
Meta-Analysis of Raw Scores on Depression Scales in Patients With Idiopathic Normal Pressure Hydrocephalus Before and After Shunt

Five studies^11,23,48,e4,e11^ reported raw scores on depressive symptom screening tools at baseline and after shunting. There were too few data to meta-analyze other symptoms. Mean symptom scores reduced after shunting by a pooled effect size of −0.30 (95% CI −0.62 to 0.01, *I*^2^: 73.9%) (Figure 4). Follow-up assessment was conducted between 3 and 4 months after shunting, except in the study^11^ where assessment of depressive symptoms was based on recall. Quality ratings were low or of some concern for all studies.

In a pilot RCT^48^ the authors found no significant treatment difference in mean BDI-II scores between placebo and true shunted cases of iNPH (−3.26, 95% CI −12.51 to 5.99), using linear regression and adjusting for potential covariates.

A study^e12^ reporting total NPI scores before and after shunting did not break these results down by symptom subtype. Participants scored a weighted mean average of 12.42 (SD 10.87) on NPI at baseline, compared with 7.61 (SD 9.50) 3 months after shunting. The reduction in NPI scores was greater among people who were AD biomarker negative.

Two studies^e4,11^ found that the prevalence of depressive symptoms reduced after shunting, from 36.0% to 5.0% on NPI and from 56.4% to 45.7% on GDS-15, respectively. One^e4^ also reported a reduction in apathy prevalence from 86.0% to 73.0%. The other study^11^ found that increased adjusted likelihood of mild (OR 5.6, 95% CI 3.6–8.8), moderate (OR 6.9, 95% CI 3.1–15.1), or severe (OR 16.2, 95% CI 3.6–74.3) depression persisted after shunting in people with iNPH compared with the general population. However, this analysis was based on recall assessment of depressive symptoms after shunting.

### Heterogeneity

Analyses with considerable heterogeneity (>75%) included depression severity score (97.9%), as well as prevalence of depression (96.3%) and psychotic syndromes (84.7%). Moderate heterogeneity was present for postshunt depression scores (73.9%), as well as prevalence of disinhibition (60.6%), agitation (51.4%), and apathy (50.5%).

To investigate heterogeneity, we performed meta-regression with age, sex, location, and diagnostic criteria for iNPH as moderators. A combined model of mean age, sex, region, and diagnostic criteria for iNPH explained most of the heterogeneity (*R*^2^: 73.57%) seen in our prevalence of depression analysis, driven primarily by region (*R*^2^: 29.54%) (eTable 3). The prevalence of depression seemed to reduce with increasing age (beta: −0.13) and to be higher in Europe (beta: 1.07). Heterogeneity in the prevalence of psychotic syndromes was fully explained by mean age and region of study (*R*^2^: 100%), with the prevalence of psychotic syndromes increasing with age (beta: 0.78). Age (*R*^2^: 27.45%) and sex (*R*^2^: 32.61%) also explained small amounts of heterogeneity for the depression severity score, with increasing age associated with reduction in effect size (beta: −0.30), though with high residual heterogeneity.

In subgroup analyses according to diagnostic criteria, the SMD between preshunt and postshunt depression scores in 4 studies using international criteria was −0.23 (95% CI −0.55 to 0.09), but there was only a single study that used Japanese criteria (−0.73, 95% CI −1.34 to −0.12) (eFigure 1). The SMD in depression scores between cases and controls was 0.94 (95% CI 0.51–1.37) in the single study that used Japanese criteria and 1.41 (95% CI 0.26–2.55) in those using international criteria (eFigure 2). The prevalence of apathy was 10% higher in international criteria studies, but otherwise, there were no discrepancies from our combined results for prevalence (eFigure 3). Subgroup analyses in higher quality studies did not differ from our pooled results.

### Sensitivity Analyses

We performed sensitivity analyses using alternative scoring cutoffs for depression in studies that reported them.^10,11,23,45,49,e1,e4^ We found that pooled prevalence reduced to 22.3% (95% CI: 17.3–28.2, *I*^2^: 71.9%) for moderate depression and to 17.5% (95% CI: 9.7–29.7, *I*^2^: 81.9%) for severe depression (eTable 4). Exclusion of the study^e1^ using the 2008 Japanese diagnostic guidelines did not change our results (eFigure 4).

Sensitivity analysis in studies with higher “certainty” of iNPH showed similar prevalence to our pooled results (eFigure 5). When considering both level of certainty and diagnostic criteria, we saw no substantial difference from our pooled results (eFigure 6). SMD of depression scores in patients with iNPH compared with controls remained large at 0.92 (95% CI: −0.01-1.86, *I*^2^: 97.5%), but CIs now contained the null (eFigure 7). Sensitivity analysis was not possible for shunt response.

## Discussion

Overall, in our review, we found that apathy was the most common neuropsychiatric finding in iNPH, with a pooled prevalence of 69.2%. Depression was also common, with a pooled prevalence of 30.1% when including mild symptoms, which dropped to 22.3% and 17.5% for moderate and severe depression, respectively. People with iNPH were much more likely to have depressive symptoms than healthy controls. Our analysis showed that shunt insertion reduced depression scores, although this effect size was small and the confidence intervals included the null. Shunting was also found to reduce overall neuropsychiatric symptom burden in 1 study. We found that people with iNPH had a higher likelihood of having any psychiatric diagnosis, schizophrenia, and depression compared with matched controls. Analysis by diagnostic criteria, study quality, and “certainty” of iNPH did not significantly alter our findings. There are few studies reporting the prevalence of neuropsychiatric symptoms in iNPH apart from apathy and depression. Most of our evidence for other symptoms came from 2 studies in Japan that used the NPI, with relatively small sample sizes. Overall, there was significant variation in psychiatric assessment tools, study design, and population used.

In comparison with other neurodegenerative conditions, we know little about neuropsychiatric features in iNPH. Despite this, in our review, we found that neuropsychiatric features may be as or more common in iNPH. In a systematic review^e13^ of neuropsychiatric symptoms in AD, apathy (49%) was lower, depression (40%) and disinhibition (17%) were similar, while anxiety (39%) and delusions (31%) were higher, compared with iNPH. One study^e1^ found that people with AD were more likely to have psychotic symptoms and agitation on the NPI when compared with iNPH. Co-pathology, particularly AD, frequently exists in iNPH. However, in this review, we did not find consistent evidence that the presence or severity of neuropsychiatric features in iNPH was explained by AD biomarker status though data were limited.

iNPH's clinical presentation is believed to be partly explained by dysfunction in subcortical brain regions such as the basal ganglia, also implicated in PD, leading to a hypokinetic disorder.^e2^ Shared neuroanatomical processes may underlie both iNPH and apathy. A previous study found reduced postsynaptic D2-receptor binding in the nucleus accumbens in patients with iNPH,^e14^ a region heavily implicated in apathy,^e15^ when compared with controls. However, 1 included study^e3 ^did not find a difference in basal ganglia dopaminergic activity between people with iNPH with and without apathy. In a review^e16^ of PD, the prevalence of apathy (40%) was lower, depression was comparable (20%–30%), and visual hallucinations were higher (22%–38%) in PD than in iNPH.

We only found 1 pilot RCT (n = 18).^48^ This did report a reduction in depression scores among people with true shunting compared with placebo, though with a wide confidence interval. One study reported a reduction in apathy score after shunting.^e4^

It is likely that the stage of iNPH influences neuropsychiatric features. Israelsson^11^ and Andersson^49^ both reported depressive symptoms in iNPH, using a cutoff of 5 and above on the GDS-15 in Sweden, but found a prevalence of 56.4% and 26.7%, respectively. Andersson et al. used a general community screening sample, while Israelsson et al. used clinically confirmed iNPH cases who were shunted. mRS scores were lower in the study by Andersson et al. compared with that by Israelsson et al. (median 2.0 vs mean 2.8). Another study^e3^ found a higher mRS score in patients with iNPH with apathy than those without apathy. Andersson et al. also found that depressive symptoms were associated with reduced quality of life. Mood reaction to functional impairment in iNPH may contribute to depressive symptoms. There was no worse severity of iNPH among those who were AD biomarker positive.^e12^

This meta-analysis explores an important and under-researched area. We performed our review according to PRISMA guidelines and undertook exhaustive analyses to ensure robust methodology and results. Despite this, our study had some limitations.

There is controversy around the proportion of people who “truly” have iNPH compared with a “degenerative” iNPH.^e17^ There may have been co-pathology driving neuropsychiatric presentation because not all studies reported on these (eg, AD biomarker status). Not all studies reported data on the stage of iNPH, which may have influenced our results. For example, hallucinations are more common in AD later in the disease process.^e18^

Studies did not include baseline assessments before iNPH, and it was not possible to assess whether the outcome of interest was present at the start of the study. These data therefore cannot indicate whether neuropsychiatric features are causally related or even associated with iNPH per se.

The methods and operationalization of symptoms differed between studies, making meta-analysis challenging. For example, our category for psychotic syndromes encompassed symptoms such as hallucinations and delusions, as well as diagnostic codes for schizophrenia, which may have lost important information.

We were unable to double screen all titles and abstracts from both searches, which may have led to some missed studies.

Significant heterogeneity remained despite meta-regression, so unexamined differences between studies existed. In addition, in 1 study, we collected prescription data for depression and psychotic disorders, but prescriptions of antidepressants and antipsychotics are indicated for a wide range of conditions and so these estimates lack specificity.

Most studies used screening instruments rather than clinical assessment. NPI was widely used but is not a diagnostic instrument.

## Conclusion

This was the first review synthesizing evidence on neuropsychiatric symptoms in iNPH. Despite the general low quality of included studies, there was a consistent finding of apathy and depression in iNPH, with a possible reduction in symptoms after treatment with shunting. Despite their significant impact on quality of life and potential treatability, very little is known about these symptoms in iNPH, as reflected by the scarcity of studies we found. Even less is known about symptoms other than apathy and depression and their response to shunting. In particular, given the high prevalence of apathy in iNPH and relative paucity of treatment, there is a need to better understand its potential response to shunting. Our study highlights the importance of further research into neuropsychiatric symptoms in iNPH, and for clinicians to consider assessment of these before and after shunting.

**TAKE-HOME POINTS**

→ We found that apathy and depression are the most prevalent neuropsychiatric features in idiopathic normal pressure hydrocephalus (iNPH), with pooled prevalence estimates of 69.2% and 30.1%, respectively.
→ Patients with iNPH demonstrate greater depressive symptom severity than controls (pooled SMD 1.31).
→ CSF shunting may reduce depressive symptoms postoperatively; however, the meta-analysis of this effect (pooled effect size −0.30) did not reach statistical significance.
→ The current evidence base is limited by low study quality and significant heterogeneity, highlighting a critical need for more methodologically rigorous research into the neuropsychiatric aspects of iNPH.

## References

[1] Hakim S, Adams RD. The special clinical problem of symptomatic hydrocephalus with normal cerebrospinal fluid pressure: observations on cerebrospinal fluid hydrodynamics. J Neurol Sci. 1965;2(4):307-327. doi:10.1016/0022-510x(65)90016-x 5889177

[2] Carswell C. Idiopathic normal pressure hydrocephalus: historical context and a contemporary guide. Pract Neurol. 2023;23(1):15-22. doi:10.1136/pn-2021-003291 36162853

[3] Toma AK, Papadopoulos MC, Stapleton S, Kitchen ND, Watkins LD. Systematic review of the outcome of shunt surgery in idiopathic normal-pressure hydrocephalus. Acta Neurochir. 2013;155(10):1977-1980. doi:10.1007/s00701-013-1835-5 23975646

[4] Pearce RKB, Gontsarova A, Richardson D, et al. Shunting for idiopathic normal pressure hydrocephalus. Cochrane Database Syst Rev. 2024;8(8):CD014923. doi:10.1002/14651858.cd014923.pub2 39105473 PMC11301990

[5] Tullberg M, Toma AK, Yamada S, et al. Classification of chronic hydrocephalus in adults: a systematic review and analysis. World Neurosurg. 2024;183:113-122. doi:10.1016/j.wneu.2023.12.094 38143036

[6] Nakajima M, Yamada S, Miyajima M, et al. Guidelines for management of idiopathic normal pressure hydrocephalus (third edition): endorsed by the Japanese Society of Normal Pressure Hydrocephalus. Neurol Med Chir (Tokyo). 2021;61(2):63-97. doi:10.2176/nmc.st.2020-0292 33455998 PMC7905302

[7] Sundstrom N, Lundin F, Arvidsson L, Tullberg M, Wikkelso C. The demography of idiopathic normal pressure hydrocephalus: data on 3000 consecutive, surgically treated patients and a systematic review of the literature. J Neurosurg. 2022;137(5):1310-1320. doi:10.3171/2022.2.jns212063 35395629

[8] Relkin N, Marmarou A, Klinge P, Bergsneider M, Black PMcL. Diagnosing idiopathic normal-pressure hydrocephalus. Neurosurgery. 2005;57(suppl l_3):S2-S16. doi:10.1227/01.neu.0000168185.29659.c5 16160425

[9] Malm J, Graff-Radford NR, Ishikawa M, et al. Influence of comorbidities in idiopathic normal pressure hydrocephalus—research and clinical care. A report of the ISHCSF task force on comorbidities in INPH. Fluids Barriers CNS. 2013;10(1):22. doi:10.1186/2045-8118-10-22 23758953 PMC3689166

[10] Valsecchi N, Mantovani P, Piserchia VA, et al. The role of simultaneous medical conditions in idiopathic normal pressure hydrocephalus. World Neurosurg. 2022;157:e29-e39. doi:10.1016/j.wneu.2021.09.071 34562629

[11] Israelsson H, Allard P, Eklund A, Malm J. Symptoms of depression are common in patients with idiopathic normal pressure hydrocephalus: the INPH-CRasH Study. Neurosurgery. 2016;78(2):161-168. doi:10.1227/NEU.0000000000001093 26528670

[12] Kanemoto H, Kazui H, Suehiro T, et al. Apathy and right caudate perfusion in idiopathic normal pressure hydrocephalus: a case-control study. Int J Geriatr Psychiatry. 2019;34(3):453-462. doi:10.1002/gps.5038 30474244

[13] Bloom KK, Kraft WA. Paranoia -- an unusual presentation of hydrocephalus. Am J Phys Med Rehabil. 1998;77(2):157-159. doi:10.1097/00002060-199803000-00012 9558018

[14] Acar M, Ozelci E, Erol A. Obsessive compulsive symptoms presented during the course of chronic normal pressure hydrocephalus: a case report. Dusunen Adam J Psychiatry Neurol Sci. 2018;31:215-221. doi:10.5350/dajpn2018310211

[15] Agrawal A, Tiwari AM, Tiple P, Chauhan MK, Nagarale M. Normal pressure hydrocephalus in a case of schizophrenia. Indian J Psychiatry 2012;54(4):385-386. doi:10.4103/0019-5545.104833 23372246 PMC3554975

[16] Paris A, Amirthalingam G, Karania T, et al. Depression and dementia: interrogating the causality of the relationship. J Neurol Neurosurg Psychiatry. 2025;96(6):573-581. doi:10.1136/jnnp-2024-334675 39798961

[17] Schrag A, Horsfall L, Walters K, Noyce A, Petersen I. Prediagnostic presentations of Parkinson's disease in primary care: a case-control study. Lancet Neurol. 2015;14(1):57-64. doi:10.1016/S1474-4422(14)70287-X 25435387

[18] Costello H, Roiser JP, Howard R. Antidepressant medications in dementia: evidence and potential mechanisms of treatment-resistance. Psychol Med. 2023;53(3):654-667. doi:10.1017/S003329172200397X 36621964 PMC9976038

[19] PROSPERO. Accessed April 9, 2025. crd.york.ac.uk/PROSPERO/view/CRD42021287293

[20] Rogers JP, Chesney E, Oliver D, et al. Psychiatric and neuropsychiatric presentations associated with severe coronavirus infections: a systematic review and meta-analysis with comparison to the COVID-19 pandemic. Lancet Psychiatry. 2020;7:611-627. doi:10.1016/S2215-0366(20)30203-0 32437679 PMC7234781

[21] WHO. The ICD-11 Classification of Mental and Behavioural Disorders: Clinical Descriptions and Diagnostic Guidelines. World Health Organization; 2018.

[22] Larsson J, Israelsson H, Eklund A, Lundin-Olsson L, Malm J. Falls and fear of falling in shunted idiopathic normal pressure hydrocephalus-the idiopathic normal pressure hydrocephalus comorbidity and risk factors associated with hydrocephalus study. Neurosurgery. 2021;89(1):122-128. doi:10.1093/neuros/nyab094 33830219

[23] Junkkari A, Häyrinen A, Rauramaa T, et al. Health-related quality-of-life outcome in patients with idiopathic normal-pressure hydrocephalus—a 1-year follow-up study. Eur J Neurol. 2017;24(1):58-66. doi:10.1111/ene.13130 27647684

[24] Mihalj M. CSF tap test - obsolete or appropriate test for predicting shunt responsiveness? A systemic review. J Neurol Sci. 2016;370:157. doi:10.1016/j.jns.2016.09.045 27772748

[25] Williams MA, Relkin NR. Diagnosis and management of idiopathic normal-pressure hydrocephalus. Neurol Clini Pract. 2013;3(5):375-385. doi:10.1212/CPJ.0b013e3182a78f6b PMC3806933 24175154

[26] Kubo Y, Kazui H, Yoshida T, et al. Validation of grading scale for evaluating symptoms of idiopathic normal-pressure hydrocephalus. Dement Geriatr Cogn Disord. 2008;25(1):37-45. doi:10.1159/000111149 18025828

[27] Banks JL, Marotta CA. Outcomes validity and reliability of the modified Rankin scale: implications for stroke clinical trials: a literature review and synthesis. Stroke. 2007;38(3):1091-1096. doi:10.1161/01.STR.0000258355.23810.c6 17272767

[28] GA Wells BS. The Newcastle-Ottawa Scale (NOS) for Assessing the Quality of Nonrandomised Studies in Meta-Analyses. The Ottawa Hospital Research Institute; 2021.

[29] Viswanathan M, Ansari MT, Berkman ND, et al. Assessing the risk of bias of individual studies in systematic reviews of health care interventions. In: Methods Guide for Effectiveness and Comparative Effectiveness Reviews. Agency for Healthcare Research and Quality (US); 2008. Accessed April 9, 2025. ncbi.nlm.nih.gov/books/NBK91433/22479713

[30] Sterne JAC, Savović J, Page MJ, et al. RoB 2: a revised tool for assessing risk of bias in randomised trials. BMJ. 2019;366:l4898. doi:10.1136/bmj.l4898 31462531

[31] Treur JL, Munafò MR, Logtenberg E, Wiers RW, Verweij KJH. Using Mendelian randomization analysis to better understand the relationship between mental health and substance use: a systematic review. Psychol Med. 2021;51(10):1593-1624. doi:10.1017/S003329172100180X 34030749 PMC8327626

[32] Luo D, Wan X, Liu J, Tong T. Optimally estimating the sample mean from the sample size, median, mid-range, and/or mid-quartile range. Stat Methods Med Res. 2018 Jun;27(6):1785-1805. doi:10.1177/0962280216669183 27683581

[33] Higgins JPT, Thomas J. Cochrane Handbook for Systematic Reviews of Interventions Version 6.3 (Updated February 2022). Cochrane; 2022.

[34] Junkkari A, Sintonen H, Nerg O, et al. Health-related quality of life in patients with idiopathic normal pressure hydrocephalus. Eur J Neurol. 2015;22(10):1391-1399. doi:10.1111/ene.12755 26104064

[35] Israelsson H, Eklund A, Malm J. Cerebrospinal fluid shunting improves long-term quality of life in idiopathic normal pressure hydrocephalus. Neurosurgery. 2020;86(4):574-582. doi:10.1093/neuros/nyz297 31504827

[36] Williams MA, Nagel SJ, Luciano MG, et al. The clinical spectrum of hydrocephalus in adults: report of the first 517 patients of the adult hydrocephalus clinical research network registry. J Neurosurgery. 2020;132(6):1773-1784. doi:10.3171/2019.2.JNS183538 31125971

[37] Hildebrandt H, Haldenwanger A, Eling P. False recognition helps to distinguish patients with Alzheimer's disease and amnestic MCI from patients with other kinds of dementia. Dement Geriatr Cogn Disord. 2009;28(2):159-167. doi:10.1159/000235643 19696484

[38] Peterson KA, Savulich G, Jackson D, Killikelly C, Pickard JD, Sahakian BJ. The effect of shunt surgery on neuropsychological performance in normal pressure hydrocephalus: a systematic review and meta-analysis. J Neurol. 2016;263(8):1669-1677. doi:10.1007/s00415-016-8097-0 27017344 PMC4971036

[39] Peterson KA, Mole TB, Keong NCH, et al. Structural correlates of cognitive impairment in normal pressure hydrocephalus. Acta Neurol Scand. 2019;139(3):305-312. doi:10.1111/ane.13052 30428124 PMC6492129

[40] Razay G, Wimmer M, Robertson I. Incidence, diagnostic criteria and outcome following ventriculoperitoneal shunting of idiopathic normal pressure hydrocephalus in a memory clinic population: a prospective observational cross-sectional and cohort study. BMJ Open. 2019;9(12):e028103. doi:10.1136/bmjopen-2018-028103 PMC6924805 31796471

[41] Lindqvist G, Andersson H, Bilting M, Blomstrand C, Malmgren H, Wikkelsø C. Normal pressure hydrocephalus: psychiatric findings before and after shunt operation classified in a new diagnostic system for organic psychiatry. Acta Psychiatr Scand. 1993;373:18-32. doi:10.1111/j.1600-0447.1993.tb05612.x 8372699

[42] De Mol J. Psychic disturbance in normal pressure hydrocephalus (author's transl). Acta Neurol Belg . 978;78(6):321-340. 749513

[43] De Mol J. Neuropsychological symptomatology in normal pressure hydrocephalus. Schweiz Arch Neurol Psychiatr Zurich Switz. 1986;137(4):33–45. . 2428104

[44] Collignon R, Rectem D, Laterre EC, Stroobandt G. Neuropsychological aspect of normal pressure hydrocephalus (author's transl). Acta Neurol Belg. 1976;76(2):74-82. 961374

[45] Koo AB, Elsamadicy AA, Lin I-H, et al. Patient risk factors associated with 30- and 90-day readmission after ventriculoperitoneal shunt placement for idiopathic normal pressure hydrocephalus in elderly patients: a nationwide readmission study. World Neurosurg. 2021;152:e23-e31. doi:10.1016/j.wneu.2021.04.010 33862298

[46] He Y, Wang Z, Zuo M, et al. The impact of neurocognitive and psychiatric disorders on the risk of idiopathic normal pressure hydrocephalus: a bidirectional Mendelian randomization study. Brain Behav. 2024;14(5):e3532. doi:10.1002/brb3.3532 38779749 PMC11112403

[47] Vanhala V, Junkkari A, Korhonen VE, et al. Prevalence of schizophrenia in idiopathic normal pressure hydrocephalus. Neurosurgery. 2019;84(4):883-889. doi:10.1093/neuros/nyy147 29741669 PMC6417909

[48] Luciano M, Holubkov R, Williams MA, et al. Placebo-controlled effectiveness of idiopathic normal pressure hydrocephalus shunting: a randomized pilot trial. Neurosurgery. 2023;92(3):481-9. doi:10.1227/neu.0000000000002225 36700738 PMC9904195

[49] Andersson J, Maripuu M, Sjövill M, Lindam A, Laurell K. Depressive symptoms, functional impairment, and health-related quality of life in idiopathic normal pressure hydrocephalus: a population-based study. PLoS ONE. 2024;19(7):e0308079. doi:10.1371/journal.pone.0308079 39078825 PMC11288432

[50] Ghaffari-Rafi A, Gorenflo R, Hu H, Viereck J, Liow K. Role of psychiatric, cardiovascular, socioeconomic, and demographic risk factors on idiopathic normal pressure hydrocephalus: a retrospective case-control study. Clin Neurol Neurosurg. 2020;193:105836. doi:10.1016/j.clineuro.2020.105836 32371292

[R51] eReferences are available as Supplementary Material at Neurology.org/cp.

